# Prevalence of Social Determinants of Health Risk Factors Among and Their Impact on Viral Suppression, Consistent Visits, and No-Show Rates Among Persons with HIV Who Identify as Hispanic

**DOI:** 10.1007/s10461-025-04630-0

**Published:** 2025-02-25

**Authors:** Mary Cowden, Ana Clavijo, Yanis Bitar, Monica Diaz, Xiomara Merced, Karla Meza, Pragnya Iyengar, Ann Avery

**Affiliations:** 1 School of Medicine, Case Western Reserve University, Cleveland, OH USA; 2 The MetroHealth System, Division of Infectious Diseases, Cleveland, OH USA; 3 Division of Infectious Diseases, The MetroHealth System, Case Western Reserve University School of Medicine, 2500 MetroHealth Dr. G533, Cleveland, OH 44109 USA

## Abstract

The current study examines the association between language preference, social determinants of health (SDOH) risk factors, viral load, and HIV care outcomes among individuals of Hispanic ethnicity in the United States. More than half (54%) of all participants reported having two or more unmet needs. Hispanic individuals were more likely to be at risk in 2 or more domains than their non-Hispanic counterparts. Food insecurity was the most common risk factor reported and when present, 51% of patients also reported financial resource strain. Surprisingly, when language preference was analyzed, English-preferring Hispanic patients were at higher risk of financial resource strain, daily stress, and food insecurity compared to those who preferred Spanish. When accounting for birthplace, Hispanic patients born in the US reported higher rates of daily stress, food insecurity, and unavailable transportation compared to Hispanic patients born in Puerto Rico or elsewhere outside the US. Increased SDOH risk factors including financial resource strain, housing and utilities strain, and limited transportation access were significantly associated with higher rates of no-shows to HIV clinic appointments but not with increased viral load or consistent completed clinic visits. In a mixed-effects model, each additional at-risk domain correlated to a 0.198 increase in the no show rate. This model also demonstrates higher rates of no-show visits in English-preferring Hispanic patients compared to Spanish-preferring patients. Overall, SDOH risk factors were commonly reported in our population and their presence were associated with higher no-show rates but not with viral suppression.

## Resumen

El presente estudio examina la asociación entre la preferencia de idioma, los factores de riesgo de los determinantes sociales de la salud (DSS), la carga viral y los resultados de la salud del VIH entre personas de etnia hispana en los Estados Unidos. Más de la mitad (54%) de todos los participantes informaron dos o más necesidades insatisfechas. Los individuos hispanos tenían más probabilidades de estar en riesgo en 2 o más dominios que sus contrapartes no hispanas. La inseguridad alimentaria fue el factor de riesgo más común informado y, cuando estaba presente, el 51% de los pacientes también informaron sobre la presión financiera. Sorprendentemente, cuando se analizó la preferencia de idioma, los pacientes hispanos que preferían el inglés tenían un mayor riesgo de escasez de recursos financieros, estrés diario e inseguridad alimentaria en comparación con los que preferían el español. Al tener en cuenta el lugar de nacimiento, los pacientes hispanos nacidos en los EE. UU. informaron tasas más altas de estrés diario, inseguridad alimentaria y transporte no disponible en comparación con los pacientes hispanos nacidos en Puerto Rico o en otro lugar fuera de los EE. UU. El aumento de los factores de riesgo de DSS, como la escasez de recursos financieros, la escasez de vivienda y servicios públicos y el acceso limitado al transporte, se asoció significativamente con tasas más altas de inasistencias a las citas clínicas para el VIH, pero no con una mayor carga viral o visitas clínicas completadas de manera constante. En un modelo de efectos mixtos, cada dominio de riesgo adicional se correlacionó con un aumento de 0,198 en la tasa de no presentación. Este modelo también demuestra tasas más altas de no presentación en pacientes hispanos que prefieren el inglés en comparación con los pacientes que prefieren el español. En general, los factores de riesgo de DSS se informaron comúnmente en nuestra población y su presencia se asoció con tasas más altas de no presentación, pero no con la supresión viral.

## Introduction

Social determinants of health (SDOH) are defined by the World Health Organization as “non-medical factors that influence health outcomes’’ [1]. These encompass a wide range of individual and community-wide factors, including income level, educational attainment, housing, and healthcare coverage, transportation access, community engagement, and discrimination. Moreover, on a broader scale, governmental systems, economic policies, climate, and geopolitical events can also significantly impact health outcomes for large groups of people.

Within the spectrum of SDOH, ethnicity plays a crucial role, encompassing factors such as culture, language, race, and nationality. For example, the presence of a language barrier between patient and provider is associated with worse healthcare outcomes and lower quality of care [2]. This is illustrated by decreased utilization of and access to screenings and preventative care, higher usage of unnecessary diagnostic testing, worse adherence with medical advice, and loss to follow up care [2, 3]. However, providers can partially mitigate this barrier by building good rapport. Patients report willingness to tolerate language barrier with providers if they “demonstrate respect and elicit trust” [4].

In addition to language, cultural concordance between patient and physician is also an important consideration. In Pandey et al., a qualitative study of the effect of English proficiency on healthcare access in Hispanics, a patient states “I am waiting to find a doctor who can speak my language and can understand my culture” [5]. Other factors unique to Hispanic culture impact care in unexpected ways. In Calo et al., a narrative study of healthcare experiences of Hispanics, Hispanic patients report frequent confusion and error impacting their medical care at pharmacies, clinics, and with insurance due to multiple surnames (a common practice in Hispanic culture but less common in the US) [6]. Race can also be an important factor, as literature has shown that Hispanic individuals with darker skin are more likely to experience color-related stigma and discrimination as well as experience worse physical health outcomes compared to those with lighter skin [7].

There has been significant research documenting the impact of SDOH on chronic diseases and HIV is no exception. In a meta-analysis of 83 observational studies with 1,445,150 patients, investigating HIV viral suppression and treatment adherence, individuals experiencing “material deprivation”- including factors such as education, employment, food insecurity, housing, income/socioeconomic status and social class- were found to have lower rates of viral suppression and adherence to antiretroviral therapy (ART). Many of the included studies demonstrated significant association between socio-economic status and viral suppression, as well as between food insecurity and ART adherence. These relationships were exaggerated in multiple marginalized groups including transgender women and unhoused/individuals experiencing housing instability [8]. Interestingly, individuals with a very low income were more likely to be virally suppressed compared to those of moderate income. This effect was theorized to be due to individuals falling below the poverty line qualifying for programs or assistance, such as the Ryan White Program [8].

The Hispanic community is disproportionately affected by HIV. According to CDC data, overall HIV incidence in the US has been declining by 6% since 2010 while HIV incidence in Hispanic populations in the US has increased by more than 14%. Annual new diagnoses of HIV in the US declined by 4% from 2012 to 2016 but increased 7% over the same period in the US Hispanic population [9]. Additionally, compared with the overall US population, Hispanics are less aware of HIV-positive status, use PrEP less frequently, and receive care for HIV at lower rates. In 2015, 60% of all HIV-positive Hispanics in the US received care for HIV, and of those individuals 20% were not retained in care for HIV [9]. The CDC estimates that less than 51% of HIV-positive Hispanic individuals in the US are currently virally suppressed [9]. Additionally, Hispanic women are greatly affected by HIV, with the rate of new HIV infections in Hispanic women 4.2x greater than in non-Hispanic white women in 2010 [10]. Cianelli et al. propose several factors that could be influencing this disparity, including many SDOH previously discussed, such as financial insecurity, barriers to access to health care and education, as well as Hispanic-specific cultural factors including Catholic tradition, which can serve as a source of community and hope for patients but also as a source of stigma such as intolerance towards LGBTQ individuals, opposition to use of condoms [10, 11]. Gender and traditional gender roles also play an important role in Hispanic culture. This is exemplified in machismo, a cultural ideal of masculinity as aggression and dominance, including engaging in risk-taking behaviors and pursuing multiple sexual partners [10, 12]. Its counterpart is marianismo, a feminine ideal of submissiveness, including prioritizing family and domesticity [10].

Some of these effects are compounded by other social factors. Previous literature suggests that country of origin is a major factor in whether Hispanic patients seek care for HIV, with Hispanics born outside the US describing a heightened fear of seeking HIV care due to concern for deportation compared to those born in Puerto Rico, who strongly identified as “not immigrant” [11].

We sought to better understand the SDOH needs of our Hispanic population living with HIV and the role language may play in reported SDOH needs and their correlation with HIV related outcomes. This data will shape programs that can more accurately target these needs and allow better allocation of resources towards things that will benefit our patients and improve their SDOH.

## Methods

All study activities were conducted at The MetroHealth System, the safety net hospital in Cleveland, OH (Cuyahoga County). The Social Determinants of Health (SDOH) Surveys used in this study are assigned in the Epic electronic health record (EHR) to patients for annual completion and was available in English and Spanish. Domains evaluated for this study included financial resource strain, food insecurity, daily stress, transportation, and housing and utilities [13–18]. Results of each domain were classified as not at risk or at risk based on the previously validated questions.

Inclusion for the study included (1) age 18 years or older (2) received HIV care at MetroHealth in last 2 years (3) Living in the greater Cleveland area (4) Living at time of analysis (5) Having HIV as one of their diagnoses on the EHR. Since the study focused on the Hispanic population specifically, every attempt was made to contact all identified Hispanic patients with HIV in the hospital system to ensure survey completion. The most recent SDOH data completed between May 2021 and February 2023 was extracted from the EHR for analysis.

To determine the patient’s preferred language while completing surveys for Hispanic patients, the study personnel would ask for the patient’s language preference. All study personnel were bilingual and therefore able to accommodate patient preference without the use of interpreter services. Study personnel also recorded the preferred language listed in the patient’s chart and noted where it differed from the stated preferred language during these conversations. Patients completing the survey via the patient portal received the questions in the language that they selected to set up in their account.

A retrospective chart review was completed for the 165 Hispanic participants who completed the SDOH survey. Viral load values and dates, total number of no show for provider visits, and attended clinic visits between June 30, 2021 to July 1, 2023 were collected. Viral load was classified as ‘Not Detectable’ if viral load was *≤* 200 copies/ml and ‘Detectable’ if > 200 copies/ml. If no viral load was completed during the time period, that record was excluded from viral suppression analyses. Only no shows with an HIV provider in the Infectious Disease Clinic were documented and counted. No show visits for any other department were not counted. Similarly, the amount of attended HIV provider visits was also tracked. Patients who had a visit with an HIV provider in each 12-month period was classified as having “Consistent follow ups”.

Statistical analyses, summaries, and tables were generated using SAS 9.4 for Windows 10. All tables were created utilizing SAS’s Proc Report function, and the p-values were computed using Fisher’s Exact Test. Table 1 examines the impact of patients’ demographic characteristics on the number of social determinants of health (SDOH) domains for which these patients are at risk.

**Table 1:** Number of Unmet social needs and participants demographic characteristics among people with HIV

Participant Characteristics		Total (*n* = 710)	0 at risk domains (*n* = 94)	1 at risk domains (*n* = 231)	2 + at risk domains (*n* = 385)	pvalue	
Age	< 30	53 (7.5%)	11 (11.7%)	15 (6.5%)	27 (7.0%)	0.000	
	30–50	306 (43.1%)	25 (26.6%)	90 (39.0%)	191 (49.6%)		
	50+	351 (49.4%)	58 (61.7%)	126 (54.5%)	167 (43.4%)		
Sex	Male	537 (75.6%)	70 (74.5%)	174 (75.3%)	293 (76.1%)	0.924	
	Female	173 (24.4%)	24 (25.5%)	57 (24.7%)	92 (23.9%)		
Race	White	294 (47.6%)	37 (45.7%)	116 (55.0%)	141 (43.3%)	0.028	
	Black	324 (52.4%)	44 (54.3%)	95 (45.0%)	185 (56.7%)		
Ethnicity	Non-Hispanic	545 (76.8%)	78 (83.0%)	188 (81.4%)	279 (72.5%)	0.013	
	Hispanic	165 (23.2%)	16 (17.0%)	43 (18.6%)	106 (27.5%)		
Language	English	638 (90.2%)	83 (89.2%)	212 (92.2%)	343 (89.3%)	0.473	
	Spanish	69 (9.8%)	10 (10.8%)	18 (7.8%)	41 (10.7%)		
Hispanic Patients Birthplace	Not United States	115 (71.0%)	14 (87.5%)	32 (74.4%)	69 (67.0%)	0.238	
	United States	47 (9.0%)	2 (12.5%)	11 (25.6%)	34 (33.0%)		
Hispanic Patients Race		Black	12 (13.3%)	1 (20.0%)	2 (7.1%)	9 (15.8%)	0.492
		White	78 (86.7%)	4 (80.0%)	26 (92.9%)	48 (84.2%)	

*Chi-squared Test*

The relationship between the SDOH domains was examined in a correlation matrix as displayed in Table 2. The correlation matrix was generated using SAS’s Proc Corr function.

**Table 2:** Correlation of social needs among hispanic persons with HIV

Pearson Correlation Coefficients Prob > |*r*| under H0: Rho = 0 Number of Observations					
	Financial Resource Strain	Housing and Utilities	Transportation	Daily Stress	Food Insecurity
**Financial Resource Strain**	1.00000 160	0.27718 0.0004 159	0.21732 0.0063 157	0.15033 0.0636 153	0.51290 < 0.0001 158
**Housing And Utilities**	0.27718 0.0004 159	1.00000 165	0.22764 0.0037 161	0.15135 0.0601 155	0.34487 < 0.0001 161
**Transportation**	0.21732 0.0063 157	0.22764 0.0037 161	1.00000 161	0.15104 0.0615 154	0.26543 0.0008 158
**Daily Stress**	0.15033 0.0636 153	0.15135 0.0601 155	0.15104 0.0615 154	1.00000 155	0.22717 0.0047 153
**Food Insecurity**	0.51290 < 0.0001 158	0.34487 < 0.0001 161	0.26543 0.0008 158	0.22717 0.0047 153	1.00000 161

Pearson correlation coefficient

**Table 3:** Unmet social needs among hispanic patients with HIV by language and birthplace

Participant Characteristics		Total (*n* = 165)	Spanish (*n* = 96)	English (*n* = 69)	*p* value	Total (*n* = 152)	Puerto Rico (*n* = 78)	United States (*n* = 47)	Other (*n* = 27)	*p* value
Financial Resource Strain	At Risk	93 (58.5%)	60 (64.5%)	33 (35.5%)	0.035	86 (58.5%)	39 (45.3%)	30 (34.9%)	17 (19.8%)	0.263
	Not at Risk	66 (41.5%)	31 (47.0%)	35 (53.0%)		61 (41.5%)	36 (59.0%)	16 (26.2%)	9 (14.8%)	
Daily Stress	At Risk	123 (79.4%)	78 (63.4%)	45 (36.6%)	0.001	115 (79.9%)	59 (51.3%)	39 (33.9%)	17 (14.8%)	0.029
	Not at Risk	32 (20.6%)	10 (31.3%)	22 (68.8%)		29 (20.1%)	18 (62.1%)	3 (10.3%)	8 (27.6%)	
Housing and Utilities	At Risk	63 (38.2%)	42 (66.7%)	21 (33.3%)	0.104	61 (40.1%)	26 (42.6%)	24 (39.3%)	11 (18.0%)	0.146
	Not at Risk	102 (61.8%)	54 (52.9%)	48 (47.1%)		91 (59.9%)	52 (57.1%)	23 (25.3%)	16 (17.6%)	
Food Insecurity	At Risk	69 (42.9%)	47 (68.1%)	22 (31.9%)	0.025	64 (43.0%)	25 (39.1%)	26 (40.6%)	13 (20.3%)	0.032
	Not at Risk	92 (57.1%)	46 (50.0%)	46 (50.0%)		85 (57.0%)	51 (60.0%)	20 (23.5%)	14 (16.5%)	
Transportation	At Risk	41 (25.5%)	24 (58.5%)	17 (41.5%)	0.857	39 (26.4%)	14 (35.9%)	19 (48.7%)	6 (15.4%)	0.014
	Not at Risk	120 (74.5%)	68 (56.7%)	52 (43.3%)		109 (73.6%)	62 (56.9%)	26 (23.9%)	21 (19.3%)	

Chi-squared Test

Table 3 explores the relationship between being at risk for financial resource strain, daily stress, housing and utilities, food insecurity, transportation, and patients’ preferred language and place of birth.

Table 4 further analyzes the relationship between being at risk for the 5 SODH domains stated in Table 3 and the number of missed doctor visits, consistent follow-ups, and detectable viral load, respectively.

**Table 4:** Prevalence and relationship between SDOH and no shows, viral suppression, and consistency of visits among hispanic patients with HIV

SDOH Domain		Total (*n* = 165)	0 no shows (*n* = 74)	1 or more no shows (*n* = 91)	*p*-value	Total (*n* = 157)	No Detected VL (*n* = 128)	Any Detected VL (*n* = 29)	*p*-value	Total (*n* = 165)	Inconsistent Visits (*n* = 68)	Consistent Visits (*n* = 97)	*p*-value
Financial Resource Strain	At Risk	93 (58.5%)	33 (35.5%)	60 (64.5%)	0.006	89 (58.9%)	70 (78.7%)	19 (21.3%)	0.530	93 (58.5%)	41 (44.1%)	52 (55.9%)	0.413
	Not at Risk	66 (41.5%)	38 (57.6%)	28 (42.4%)		62 (41.1%)	52 (83.9%)	10 (16.1%)		66 (41.5%)	24 (36.4%)	42 (63.6%)	
Daily Stress	At Risk	123 (79.4%)	52 (42.3%)	71 (57.7%)	0.320	116 (78.9%)	98 (84.5%)	18 (15.5%)	0.192	123 (79.4%)	55 (44.7%)	68 (55.3%)	0.108
	Not at Risk	32 (20.6%)	17 (53.1%)	15 (46.9%)		31 (21.1%)	23 (74.2%)	8 (25.8%)		32 (20.6%)	9 (28.1%)	23 (71.9%)	
Housing and Utilities	At Risk	63 (38.2%)	22 (34.9%)	41 (65.1%)	0.054	60 (38.2%)	48 (80.0%)	12 (20.0%)	0.833	63 (38.2%)	27 (42.9%)	36 (57.1%)	0.747
	Not at Risk	102 (61.8%)	52 (51.0%)	50 (49.0%)		97 (61.8%)	80 (82.5%)	17 (17.5%)		102 (61.8%)	41 (40.2%)	61 (59.8%)	
Food Insecurity	At Risk	69 (42.9%)	26 (37.7%)	43 (62.3%)	0.150	67 (43.8%)	54 (80.6%)	13 (19.4%)	1.000	69 (42.9%)	31 (44.9%)	38 (55.1%)	0.420
	Not at Risk	92 (57.1%)	46 (50.0%)	46 (50.0%)		86 (56.2%)	70 (81.4%)	16 (18.6%)		92 (57.1%)	35 (38.0%)	57 (62.0%)	
Transporta-tion	At Risk	41 (25.5%)	11 (26.8%)	30 (73.2%)	0.007	39 (25.5%)	29 (74.4%)	10 (25.6%)	0.147	41 (25.5%)	21 (51.2%)	20 (48.8%)	0.202
	Not at Risk	120 (74.5%)	62 (51.7%)	58 (48.3%)		114 (74.5%)	97 (85.1%)	17 (14.9%)		120 (74.5%)	47 (39.2%)	73 (60.8%)	

Chi-squared Test

The influence of preferred language, sex at birth, and the number of at-risk SDOH domains on the number of no-show provider visits among Hispanic patients was assessed using a linear mixed-effects model, as presented in Table 5. The model was constructed using SAS’s Proc Mixed function. An alpha level of 0.05 was employed to determine statistical significance in the analysis.

**Table 5:** Mixed effects Model of the relationship between domains at risk and probability of no shows among hispanic patients with HIV

Effect	Class	Estimate	StdErr	DF	tValue	Probt
Intercept		0.5270	0.3079	161	1.71	0.0889
Domains at Risk		0.1858	0.09196	161	2.02	0.0450
Preferred Language	Spanish	0.00	.	.	.	.
	English	0.5672	0.2857	161	1.99	0.0488
Sex	Male	0.00	.	.	.	.
	Female	0.0569	0.2889	161	0.20	0.8441

Linear mixed model analysis

All study activities were approved under local IRB ID: STUDY00000101D.

## Results

Overall, there were 711 completed surveys with 165 of those from Hispanic individuals. In the same period, there were 1465 individuals who received HIV care at that site, 212 who identified as Hispanic yielding a 48.5% overall completion rate and 78% for Hispanic individuals. Among Hispanic individuals, reasons for not completing including being unreachable, active declination, relocated, changed providers, or were incarcerated at the time of survey. Participant demographic characteristics are provided in Table 1. In general, participants reported a high prevalence of social domains at risk. More than half (54%) of the total participants reported 2 or more at risk domains, while only 13% reported 0 at risk domains. Individuals ages 30–50 had the highest rate of 2 + domains at risk. Hispanic individuals were less likely to have 0 domains at risk and more likely to have 2 + domains at risk than non-Hispanic individuals. Of those who identified as Hispanic, only 12 (13.3%) also identified as Black, the remainder were evenly split between identifying as white or declining to/ not selecting a race category. In Table 2, the correlation matrix among Hispanic individuals revealed food insecurity to exhibit the strongest correlation with all other domains. Specifically, if a Hispanic individual reported food insecurity, there was a 51% likelihood of also experiencing potential financial insecurity.

When SDOH results for Hispanics were analyzed by preferred language, more English-preferred speaking Hispanic patients identified as being at-risk for financial resource strain (*p* =.035), daily stress (*p* =.001) and food insecurity (*p* =.025) compared to Spanish-preferred speaking Hispanic patients (Table 3). Additionally, SDOH results were analyzed by birthplace, and US born Hispanics had higher rates of transportation strain (*p* =.014), daily stress (*p* =.029) and food insecurity (*p* =.03) compared to Puerto Rican and foreign-born Hispanics while the other domains were not significant. There were no significant differences between males and females.

## Impact on HIV Outcomes

To better understand the impact of SDOH on HIV related health outcomes in the Hispanic population, we examined the relationship between domains at risk and no shows, viral suppression, and the consistency of visits (Table 4). We found that participants who reported being at risk for financial resource strain (*p* =.006), housing and utilities (*p* =.054), and transportation (*p* =.007) were more likely to have 1 or more no shows in the study period. Interestingly, no significant differences in completed visits or viral suppression was noted in univariate analyses.

The mixed-effects model (Table 5) adjusted for sex, number of SDOH domains at risk and preferred language spoken. It showed that English-speaking Hispanic patients are estimated to have 0.567 ± 0.286 (*p* =.049) more no-shows than Spanish-speaking Hispanic patients. Furthermore, the model showed that for every additional SDOH domain at risk the number of no shows increased by 0.186 ± 0.092 (*p* =.045).

## Discussion

To our knowledge this is the first comprehensive assessment evaluating the association between language preference, birthplace, SDOH risk factors, viral load, no-show rates, and consistent visit patterns among individuals of Hispanic descent in the United States. Our results revealed a notable prevalence of unmet social needs, with more than half (54%) of all participants reporting having two or more unmet needs. The most common unmet need among Hispanics was daily stress (79.4%) followed closely by food insecurity (58.5%) and financial stress (42.9%). This observation aligns with existing literature, underscoring that ethnic minorities disproportionately face higher levels of social risk factors compared to White individuals [19–21].

Surprisingly, we found that SDOH risk factors did not have a significant impact on viral load or consistent clinic visits. We had anticipated a relationship between increased risk factors and worsening viral loads because of a recent study documenting increased SDOH risk factors and decreased anti-retroviral therapy adherence among a sample of Black people with HIV [22]. However, we did observe that as the number of SDOH risks increased, so did the likelihood of no-shows. This aligns with the growing body of literature across multiple specialties showing that unmet social needs, Medicaid or no insurance coverage, lower socio-economic status, and new patient visits (as opposed to established patients) are all predictors of missed appointments [23–26]. Fortunately, we did not observe an impact on viral load or the consistency of clinic appointments. Thus, although patients confronting heightened social barriers demonstrated increased rates of missed appointments, these challenges did not necessarily influence their viral load levels. We suspect this is due to the widespread understanding of the importance of medication adherence, newer medications that may be more effective, and minimal barriers for our patients to get medications.

No-shows may reflect the chaotic lifestyle of persons reporting SDOH risks; attending a previously scheduled appointment when one is ‘doing fine’ may be less important than addressing other basic human needs. Current guidelines recommend that HIV-positive patients see their provider every 6 months, however this may not be feasible for patients with social barriers and may contribute to an increase in no show rates if they can’t attend the appointments as often as they’re being scheduled [27]. Like our study suggests, financial strain, housing difficulties, and lack of transportation all led to increased likelihood of no-show rates among our population but there are many other reasons HIV-positive patients report nonadherence to appointments. Recent studies specific to HIV clinics also report barriers due to geographic distance between the healthcare facility and patient’s housing, and personal life circumstances such as substance use, mental health, homelessness, or fear of HIV disclosure [28, 29]. Our study and supporting literature suggest that no show reasons are multi-factorial and addressing the root problem would likely need an integrated approach.

No shows are an important topic because they are a wasteful economic burden to the US healthcare system. A retrospective review from the Veterans Health Administration documented that for 10 clinics the cost of no-shows in 2008 was $14.58 million [30]. However, more recent reports estimate that yearly US healthcare spending for no shows is $50 billion [31]. In addition to the economic burden for the health care system, on a smaller scale it also creates dilemmas for prospective patients trying to schedule due to the absence of timely and available appointments [32]. These burdens are problematic from a healthcare system standpoint, and we could be using these funds productively for public health interventions to target the root of the no-show. Additional strategies for reducing no-show rates have shown promise in other specialty settings, such as use of an Open-Access schedule, language concordance between provider and patient, decreased lead time before appointments, and geographic proximity to the clinic [23]. In one study, availability of tele-health was effective at reducing no-show rate in socially vulnerable patients; however, in another study, tele-health visits were shown to have higher rates of no-show than in-person encounters [24, 25]. **Our findings suggest that addressing unmet needs may help to improve no-show rates.** Given the limited understanding of these factors based on current studies, especially in HIV-specific settings, further research needs to be done to better measure no-show rates, evaluate additional factors that influence them, and develop strategies to address these issues.

It is still crucial to implement strategies to help patients adhere to ART even if they miss their appointments. An international literature review identified multiple studies that trialed strategies to increase adherence [33]. While there have previously been several methods explored, pill alarms, text messages, weekly reminders, and community health workers, these have not actually led to improved ART adherence. The review did identify five studies that successfully increased adherence to ART. All of these studies included some version of intensive behavioral counseling in their intervention arm [34–38]. Therefore, implementing more in-depth counseling during appointments when the patients do come may help adherence to ART and mitigate the potentially negative health impact of missing appointments.

We also observed that food insecurity had the most pronounced correlation with all other domains and served as a predictor for potential financial insecurity. A review article noted that food insecurity plays a very important role in HIV infection. It both exacerbates vulnerability to HIV infection and worsens clinical outcomes [39]. **Therefore, public health interventions focused on decreasing food insecurity among these vulnerable populations are extremely important to target the other domains as well.**

When we analyzed the results by language preference, English-speaking Hispanic patients displayed the highest prevalence of almost all social needs (financial insecurity, daily stress, food insecurity, transportation, housing & utilities) compared to their Spanish-speaking counterparts. This finding contradicted our initial hypothesis. It also contradicted our review of other literature that documents language barriers contributing to decreased access to care, underutilization of care, limited comprehension of their medical conditions, challenges in securing well-paid employment, difficulties accessing food, and limited awareness of community resources [19, 40–43]. However, there is a current gap in research regarding the prevalence of social determinants of health categorized by language preference. Therefore, our study assumes significance in addressing this gap and contributing to the understanding of these factors.

While we initially expected US and Puerto Rican born patients to have fewer social needs due to stable immigration status, our results differ from this initial hypothesis. A patient’s rationale for immigration can influence socioeconomic status and appropriate access to healthcare [44]. For example, Puerto Ricans who originally immigrated to the US during the 1950s due to economic change and need for new opportunity were found to be of lower socioeconomic status (SES) while many Cubans who immigrated for political reasons often had a higher SES [44]. Preexisting wealth, social support, and other resources can influence patients’ current social needs. US-born Hispanics experience racism and discrimination from an early age; studies among the Black population in the United States show a connection between perceived discrimination and poor physical and mental health [45, 46]. This could explain the greater social needs reported by US-born Hispanic patients, especially greater daily stress. Concurrently, studies have shown that Hispanic patients born outside of the US were more likely to be prescribed ART, engage in consistent follow-up, and sustain viral suppression [47, 48]. One explanation for this observation is that Hispanic patients born outside the US are more likely to present with more advanced disease; a study found non-US and PR-born patients are more likely to have AIDS and more than 50% of Hispanic patients born outside the US received their diagnoses over 10 years after immigrating [47]. This could serve as an important motivator to adhere to medication regimens and careful follow-up.

We hypothesize that the language and birthplace associations with less SDOH needs we found could be attributable to a “healthy immigrant” effect, in that potentially the Spanish-speaking Hispanic population in our survey included those who recently immigrated to the United States and might be overall healthier individuals with less social factors at risk [49]. Another plausible explanation could be that primarily Spanish-speaking Hispanics may be less “accultured” and therefore have stronger familial ties to rely on for social assistance. This is a concept known as “familismo” which refers to a cultural obligation to provide family members with emotional support or assistance in times of need. In the literature, familismo has been perceived as a major positive factor for overall health and health behaviors. It has been documented linking to positive health outcomes including lower rates of substance and drug abuse [50, 51].

Our findings should be interpreted in the context of a few study limitations. The self-reported nature of the survey responses might be underestimated due to desirability bias and discomfort. Additionally, individuals undergoing immigration proceedings might receive legal advice against seeking healthcare unless prepared to cover expenses out of pocket, potentially affecting accuracy of reported data. It is noteworthy that most Hispanic patients in our study are Puerto Rican born and therefore may not face the same immigration concerns that other Hispanics may while seeking healthcare. This potentially limits comparisons between Hispanic and non-Hispanic groups. Completion rates within the general clinic were also lower, which impacts the generalizability of our findings.

Lastly, we performed this study at an urban, safety net hospital where several of the HIV clinic providers speak both English and Spanish. Patients also have access to bilingual social workers at every visit for in-person assistance with transportation, medication access, and insurance concerns. Therefore, it is possible that language barriers were not necessarily a barrier to receiving care in our study compared to other locations that might not have bilingual care providers.

## Conclusion

In conclusion, our results reveal that Hispanic patients are more likely to have SDOH risk factors compared to their non-Hispanic counterparts, and though this increased SDOH risk is correlated with a higher rate of no-shows at HIV appointments, it does not correlate with an increase in viral load. Further research is needed to better understand the relationship between viral load, SDOH risk factors, and retention in HIV care. This includes additional investigation into interventions designed to reduce the no-shows and address SDOH risk factors. 
